# Hepatitis C missed diagnostic opportunities: The role of reflex viral load testing in the era of direct acting antivirals, a descriptive South African study from 2017 to 2021

**DOI:** 10.4102/sajid.v41i1.778

**Published:** 2026-03-27

**Authors:** Hloniphile Mthiyane, Bonolo Mashishi, Mamodiane A. Patjane, Ntwanano Honwana, Nishi Prabdial-Sing, Lucia Hans, Nonhlanhla Mbenenge, Bhaveshan Reddy, Avania Bangalee, Florette K. Treurnicht

**Affiliations:** 1 Department of Medical Virology, Faculty of Health Sciences, University of the Witwatersrand, Johannesburg, South Africa; 2 Department of Medical Virology, Faculty of Health Sciences, University of Pretoria, Pretoria, South Africa; 3 Department of Medical Virology, National Health Laboratory Service, Johannesburg, South Africa; 4 National Institute for Communicable Diseases, National Health Laboratory Service, Johannesburg, South Africa; 5 National Priority Programme, National Health Laboratory Service, Johannesburg, South Africa; 6 Wits Diagnostic Innovation Hub, Faculty of Health Sciences, University of the Witwatersrand, Johannesburg, South Africa; 7 Department of Medical Virology, Lancet Laboratories, Johannesburg, South Africa

**Keywords:** HCV, serology, viral load, missed diagnoses, testing, public sector

## Abstract

**Background:**

The hepatitis C virus (HCV) testing algorithm used in South African laboratories involves two tests; a serology test to detect anti-HCV antibodies and a confirmatory test to detect the presence of HCV ribonucleic acid (RNA). These tests usually require consecutive blood samples taken at more than one hospital visit, leaving patients with inconclusive diagnosis for weeks.

**Objectives:**

This study aimed to describe the prevalence and proportion of HCV seropositive cases where diagnostic opportunities were missed among patients who attended public healthcare facilities in Johannesburg, South Africa.

**Method:**

This descriptive study was conducted as a retrospective analysis of laboratory data from the Department of Medical Virology, National Health Laboratory Service, Johannesburg, South Africa. Patients for whom HCV diagnosis were requested from 2017 to 2021 were included in the study.

**Results:**

Over the five-year period, a total of 212 670 patients were tested for HCV antibodies and 8575 (4.04%) tested positive. The HCV seropositivity was recorded for 74.8% (6421/8575) of males compared to females (23.6%; 2030/8575; *p* < 0.001). Only 25% (2183/8575) of HCV seropositive patients had a linked HCV RNA quantitative PCR test result.

**Conclusion:**

Despite an overall HCV seroprevalence of 4% in this study, only one quarter received a definitive diagnosis. This highlights that most patients are lost to follow-up because of the two-step diagnostic process, which requires them to return to the health facility for confirmatory tests.

**Contribution:**

The study highlights the need to adopt HCV reflex testing as the standard in South Africa to meet the 2030 elimination targets.

## Introduction

In 2022, it was estimated that 50 million people were chronically infected with hepatitis C virus (HCV), with an average of just over a million new infections occurring annually.^1^ As a result, approximately 242 000 people succumbed to the infection, especially those with long-term complications.^1^ A subset of infected individuals (15% – 25%) will spontaneously clear the virus, while 75% – 85% will progress to chronic infection with an increased risk of developing chronic liver cirrhosis and hepatocellular carcinoma.^2^ Acute hepatitis C infection is predominantly asymptomatic, with 20% of cases presenting with mild symptoms such as fatigue, nausea, vomiting, diarrhoea and dark urine.^2,3^ Consequently, to accurately confirm the diagnosis of HCV infection laboratory-based testing comprised of both serological and molecular techniques is necessary.^3^ In the era of curable treatment for HCV, early and accurate HCV diagnosis is key to meeting the World Health Organization (WHO) elimination goals for 2030, which aim for the reduction of new HCV infections by 90% and mortality by 65%.^1,3^ Post-HCV infection, HCV ribonucleic acid (RNA) is usually detectable in serum after one to three weeks. Detectable HCV RNA in an individual’s sera beyond 6 months indicates a persistent chronic infection.^4^ Curative therapy had been available for HCV and can achieve sustained virological response (SVR) in 30% – 50% of patients; however, many individuals experienced significant adverse effects.^5^ In 2022, direct acting antivirals (DAA) therapy was approved for use in South Africa, following a lengthy clinical observation for effectiveness and tolerance, marking a major milestone in HCV treatment and management.^6,7^ However, access to DAAs has remained limited because of high costs and limited diagnostic infrastructure.^6,7^ The DAAs aim to clear the virus and lessen the chance of severe disease progression. Successful treatment is measured by a SVR with undetectable HCV RNA 12 weeks after treatment is stopped.^8^ The effectiveness of DAA therapy is further enhanced by the use of pan-genotypic agents.^6,9^ South African seroprevalence data show that anti-HCV is approximately 1% or less in the general population and varied among high-risk groups.^6,7,10^ The accuracy and validity of overall HCV prevalence may be compromised because of poor follow-up and linkage to care.^8^

The HCV testing algorithm used in South African laboratories involves two tests: an initial serological test to detect anti- HCV antibodies and if positive, followed by a confirmatory test to detect the presence of HCV viral RNA.^8^ A significant drawback with the previous diagnostic algorithm was the need for additional hospital visits weeks later, where a second sample was extracted for HCV quantitative RNA PCR to confirm and describe viral load (VL). The testing required screening and confirmatory samples from more than one hospital visit, consequently, patients were left with an inconclusive diagnosis for weeks.^8^ Recently, a laboratory-based HCV reflex testing protocol was developed by the Department of Health to counteract inconclusive results and loss of follow-up.^8^ Laboratory-based HCV reflex testing refers to a testing algorithm in which patients have only a single clinical encounter and one blood draw or specimen for an initial laboratory-based HCV antibody test, which is then sent to the laboratory.^11^ If the sample for HCV antibody testing is positive, then the same sample is automatically used for a prompt ‘reflex’ laboratory-based HCV RNA test.^8,11,12^ The result returned to the patient or medical practitioner is for both the HCV antibody and HCV RNA results. Therefore, no further visit or specimen collection is required.^8,12^

### Objectives

This study described: (1) the seroprevalence of HCV from samples submitted for testing in a public sector, Gauteng in South Africa from 2017 to 2021; (2) the proportion of HCV antibody-positive patients who received or did not undergo a follow-up HCV RNA PCR test to diagnose viraemia from 2017 to 2021.

## Research methods and design

### Study design

This is a cross-sectional retrospective descriptive study that used routine laboratory data from Gauteng, South Africa. Criteria for inclusion in this study were all patients for whom HCV diagnostic testing were requested from the National Health Laboratory Service (NHLS) Virology Laboratory at Charlotte Maxeke Johannesburg Academic Hospital (CMJAH), from 01 January 2017 to 31 December 2021. During this study period the Elecsys Anti-HCV II assay (Roche Diagnostics, Mannheim, Germany) processed on the automated Roche Cobas e602 analyser was used to detect antibodies to HCV in human serum and plasma samples. The anti-HCV serology results were interpreted as follows; (absorbance values categorised as negative [< 0.9], equivocal [> 0/9 – < 1], low positive [≥ 1 – < 10] and positive [≥ 10]). Hepatitis C virus viral RNA was determined using the COBAS AmpliPrep/COBAS TaqMan HCV Test (Roche Molecular Systems, New Jersey, United States) for automated nucleic acid extraction followed by amplification and detection on the COBAS TaqMan 96 analyzer. Hepatitis C virus viral RNA results are reported in international units per millilitre (IU/mL), with ≤ 15 IU/mL as a limit of detection.

All anti-HCV serology and HCV RNA results were extracted from the NHLS Corporate Data Warehouse (CDW), the central data repository for laboratory results within the public health sector in South Africa.

### Data collection and statistical analysis

Data were extracted from the NHLS CDW as Excel spreadsheet and imported into Statistica software version 14.0.0.10 for analysis; *p* ≤ 0.05 was considered statistically significant. The variables included in the analysis were age, sex, hospital admission, anti-HCV serology results and HCV viral RNA results. Hepatitis C virus viral RNA results were categorised as individuals with or without HCV RNA test. Among those with HCV RNA test, results were further categorised as viraemic (> 15 IU/mL) or below the limit of detection. Descriptive statistics were used to summarise the data; categorical variables were presented as frequencies and proportions. While non-parametric continuous variable was described using median and interquartile ranges at a 95% confidence interval (CI). Box whisker plot with the Kruskal–Wallis test was used for analysis of the distribution.

### Ethical considerations

Ethical clearance was obtained from the Human Research Ethics Committee of the University of Witwatersrand (reference number: M201074). Data were extracted from the NHLS CDW as a Microsoft Excel spreadsheet. Patient identifiers were anonymised, and the password-protected file was only accessible to the principal investigator and the main researcher. All data were securely stored in accordance with ethical research guidelines and confidentiality standards.

## Results

### Overall hepatitis C virus serology and nucleic acid testing

**FIGURE 1:**
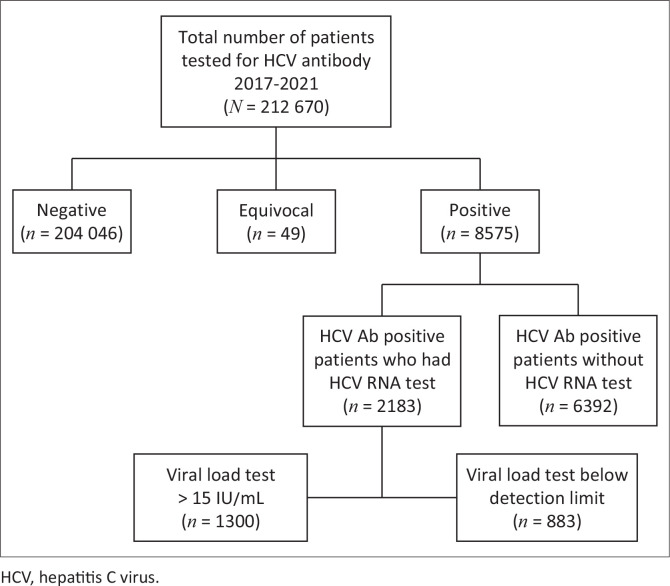
Flow diagram indicating the number of sample sets from collection to analysis.

A total of 212 670 patients were tested for HCV infection from 01 January 2017 to 31 December 2021 at NHLS (Figure 1). A total of 8575 (4.04%) tested seropositive for HCV. Low positives were overall present at 0.20% (*n* = 427/212 670) and represent 5.0% (*n* = 427/8575) of HCV seropositive cases (Table 1). A linked follow-up quantitative HCV RNA test was performed for 25% (*n* = 2183/8575) of which 59.55% (*n* = 1300/2183) had detectable viral RNA (Figure 1).

**TABLE 1:** A summary of hepatitis C virus serology results among patients from public health facilities in Johannesburg, South Africa, 2017 to 2021 (*N* = 212 670).

Variable	2017		2018		2019		2020		2021		Total	
	*n*	%	*n*	%	*n*	%	*n*	%	*n*	%	*n*	%
Negative	46 604	22.8	45 227	22.3	44 581	21.8	37 817	18.5	29 817	14.6	204 046	95.94
Equivocal	1	2.0	6	12.0	8	16.0	12	25.0	22	45.0	49	0.02
Total positive	1709	20.0	1557	18.1	2072	24.1	1821	21.3	1416	16.5	8575	4.04
Positive	1611	19.7	1517	18.6	1977	24.2	1706	20.9	1337	16.6	8148	3.84
Low positive	98	22.9	40	9.3	95	22.4	115	26.9	79	18.5	427	0.20

**Total**	**48 314**	**22.7**	**46 790**	**22.2**	**46 661**	**21.9**	**39 650**	**18.6**	**31 255**	**14.6**	**212 670**	**100**

### Annual variations in hepatitis C virus serology

Of the 212 670 patients who were tested for HCV infection, the highest numbers for HCV serology were tested in 2017 (22.7%; *n* = 48 314), 2018 (22.2%; *n* = 46 790), and 2019 (21.9%; *n* = 46 661) at similar rates (Table 1). In 2020, fewer patients were tested and represented 18.6% (*n* = 39 650/212 670), while 2021 only represented 14.6% (*n* = 31 255/212 670). The annual HCV antibody positivity rate was lowest in 2021 at 16.5% (*n* = 1416/31 255) and was 21.3% (*n* = 1821/39 650) in 2020. The highest HCV antibody positivity rate of 24.1% (*n* = 2072/46 661) was observed in 2019.

### Demographic and clinical characteristics of hepatitis C virus seropositive patients

Of the 8575 HCV-seropositive patients, males were significantly higher than females (*n* = 6421, 74.8% vs *n* = 2030, 23.6%; *p* < 0.001), while for 1.5% (*n* = 124/8575) sex was not recorded (Table 2).

**TABLE 2:** Demographic characteristics of hepatitis C virus seropositive patients (*N* = 8575).

Variable	2017				2018				2019				2020				2021				Total				*p*-value
	*n*	%	Median	IQR	*n*	%	Median	IQR	*n*	%	Median	IQR	*n*	%	Median	IQR	*n*	%	Median	IQR	*n*	%	Median	IQR	
**Sex**	-	-	-	-	-	-	-	-	-	-	-	-	-	-	-	-	-	-	-	-	-	-	-	-	< 0.001*
Male	1191	18.5	-	-	1128	17.5	-	-	1605	24.9	-	-	1384	21.5	-	-	1113	17.3	-	-	6421	74.9	-	-	-
Female	490	24.1	-	-	403	19.8	-	-	440	21.6	-	-	411	20.2	-	-	286	14.0	-	-	2030	23.6	-	-	-
Unknown	28	22.5	-	-	26	20.9	-	-	27	21.7	-	-	26	20.9	-	-	17	13.7	-	-	124	1.5	-	-	-
**Age (years)**	-	-	-	-	-	-	-	-	-	-	-	-	-	-	-	-	-	-	-	-	-	-	-	-	0.062
0–12	206	-	0	0–0	134	-	0	0–0	58	-	0	0–0	123	-	0	0–0	101	-	0	0–0	722	-	0	0–0	-
13–19	41	-	18	17–19	37	-	18	17–19	33	-	18	16–19	13	-	18	16–19	14	-	18	16–19	138	-	18	17–19	-
20–35	769	-	27	25–31	749	-	28	25–31	1191	-	28	25–31	985	-	29	26–32	757	-	29	26–31	4451	-	28	25–31	-
36–54	346	-	42	38–48	309	-	42	38–49	384	-	41	38–46	417	-	40	38–46	345	-	40	37–45	1801	-	41	38–47	-
55–70	253	-	63	58–66	213	-	62	59–66	206	-	63	59–67	195	-	62	58–67	152	-	64	59–67	1019	-	53	59–67	-
> 71	94	-	76	73–79	115	-	77	73–81	100	-	76	73–80	88	-	75	73–81	47	-	75	72–81	444	-	76	73–80	-
**Hospital admission**	-	-	-	-	-	-	-	-	-	-	-	-	-	-	-	-	-	-	-	-	-	-	-	-	< 0.002*
Yes	896	19.7	-	-	892	19.8	-	-	1067	23.5	-	-	946	21.2	-	-	703	15.6	-	-	4504	52.6	-	-	-
No	813	19.9	-	-	665	16.3	-	-	1005	24.6	-	-	875	21.4	-	-	713	17.5	-	-	4071	47.4	-	-	-

IQR, interquartile range.

* , Statistically significant.

**FIGURE 2:**
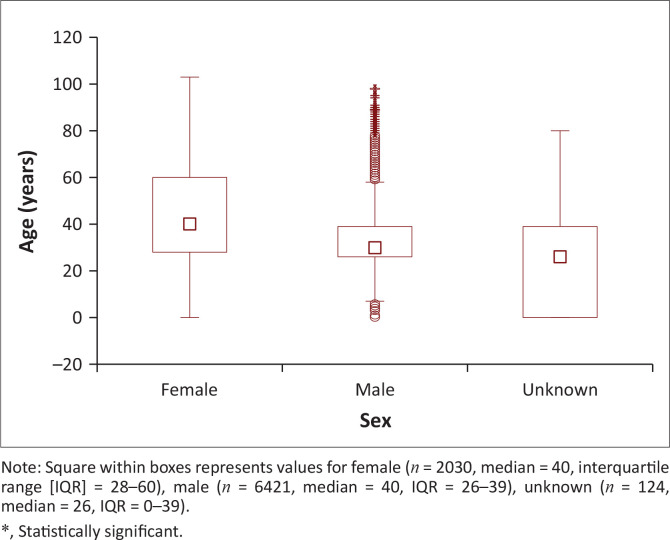
Box and whisker plot illustrating age distr ibution of hepatitis C virus seropositive patients by sex using Kruskal–Wallis test (*N* = 8575, *p* < 0.001*).

Males were also significantly younger than females (median age 30 [26–39] years vs 40 [28–60] years; *p* < 0.001) (Figure 2). The year 2019 reported the highest number of males, while 2018 reported the lowest number of females. Among HCV seropositive patients, the age group 20–35 years with a median age of 28 years old dominated (52%; *n* = 4451/8575), and seropositivity was the highest in 2019, (26.7%; *n* = 1191/4451), followed by age groups 36–54 years (21%; *n* = 1801/8575) and 55–70 years (12%; *n* = 1019/8575). Among children 12 years and younger HCV seropositivity was 8.4% (*n* = 722/8575) with the lowest seropositivity observed among the 13–19 years age group (median 18 years).

The overall number of HCV-seropositive patients with hospital admission (*n* = 4504/8575, 52.6%) was significantly higher compared to those without hospital admission (*n* = 4071/8575, 47.4%; *p* < 0.002) (Table 2). The highest number of admissions was reported in 2019 (23.5%), whereas the lowest was reported in 2021 (15.6%) (Table 2).

## Discussion

Previously in South African practice, the diagnosis of HCV required at least two visits to screen and diagnose HCV infection; as a result, a proportion of patients will be lost to follow-up and they could not be linked to care.^8,14^ Improving access to rapid, simple and affordable HCV diagnostics is critical to advancing global HCV elimination efforts. In 2019, a laboratory-based HCV reflex testing algorithm was introduced to address issues related to inconclusive results and patient loss to follow-up.^8^ This study included 212 670 patients tested for HCV antibodies over 5 years in the public sector, making this one of the largest HCV descriptive studies focusing on a general South African population. Of the 212 670 patients serologically tested for HCV antibodies, 204 046 (95.9%) tested negative, while 8575 (4.04%) tested seropositive. In this study, we are reporting 4.04% seroprevalence from a general South African cohort. Furthermore, the seroprevalence observed in our study is considerably higher than previous estimates of less than 1% for South Africa’s general population and higher than the 2.64% reported for the general population in sub-Saharan Africa.^6,7,11,12,13,14^ Research describing HCV seroprevalence in South Africa has largely been directed to the high-risk populations, including people who inject drugs (PWID), men who have sex with men (MSM), HCV and HIV concurrently infected patients, the homeless, and the incarcerated because of their high HCV prevalence rates.^11,12,13,14^ The accurate seroprevalence data in a broader population is necessary for South Africa to track progress and refine elimination strategies towards the WHO’s target of eliminating HCV as a public health threat by 2030.^1^ Our findings, drawn from a large public sector cohort tested over five years, suggest a potentially broader transmission of HCV within the general population. This highlights the urgent need for ongoing reassessment and updated prevalence studies to inform national elimination efforts. The HCV RNA testing was performed for only 25% of seropositive patients, among whom more than half had detectable HCV RNA copies ranging between < 15 IU/mL and < 10 000 000 IU/mL. Our findings are consistent with other studies conducted in South Africa and across sub-Saharan Africa, which also report that over half of HCV-seropositive individuals were viraemic.^12,14,15^ However, what is particularly concerning is that in our study, a staggering 75% of seropositive patients did not have a linked HCV RNA result. This finding substantiates our hypotheses that the HCV diagnostic algorithm recommended by the WHO and by the South African Hepatitis National Guidelines are not adhered to in clinical practice. Failure to confirm active HCV infection delays crucial interventions, such as timely initiation of treatment in the DAA era, thereby prolonging disease progression and increasing the risk of complications.^6,16^

Furthermore, undiagnosed individuals with detectable HCV RNA may continue to transmit the virus especially among high-risk groups and at a population-based level.^16^ This ongoing transmission could sustain high rates of infection, undermining efforts to achieve the WHO’s target of a 90% reduction in new HCV infections by 2030. A significantly higher proportion of males (74.8%) were found to be seropositive than females (23.6%), and younger across all 5 years.

These results correlate with several studies conducted on HCV prevalence.^5,17^ Since the early 2000s, sexual transmission has been recognised as an emerging risk factor for HCV infection, particularly among HIV-positive MSM.^18^ The risk is further compounded by concomitant injecting drug use in this population.^19^ We observed a similar distribution across all age groups, with the majority of cases occurring among young adults, with a median age of 28 years (interquartile range [IQR]: 25–31), representing the highest number of HCV-seropositive results. This observation is widely reported in the literature and is often attributed to the higher representation of males in high-risk groups such as PWID, MSM and incarcerated individuals.^5,20^ Other studies have also reported a notable rise in HCV cases among individuals aged 20–29 years, which were primarily linked to injection drug use and high-risk behaviour.^11,12,13,14^ Also, higher prevalence among young adults emphasises the role of intravenous drug use in the transmission of HCV infection.The risk of transmission of HCV among young children born to HCV-infected mothers is approximately 5%. This risk increases to approximately 10% in mothers co-infected with HIV or with HCV viral loads ≥ 10^6^ IU/mL.^21,22^ Of the 8575 HCV-seropositive patients, 722 (8.4%) were children younger than 12 years of age, with a median age of 0 years. A reported 25% to 40% of children with perinatally acquired HCV infection may spontaneously clear the virus, usually between the ages of 2 years and 5 years,^23,24^ which may explain the observed decline of HCV infection in the 13–19 year age group, where the median age was 18 years and prevalence was 1.2%. In this study period, hospital admissions were overall significantly higher compared to outpatients (*p* < 0.002), although this occurrence may not be directly linked to HCV infection. Over the years, observational studies conducted in high-income countries have shown that among HCV infected individuals, hospitalisation related to HCV infection was gradually increasing. However, since the introduction of DAAs, hospitalisation rates have markedly declined, thereby reducing the overall healthcare burden.^25,26^ In contrast, our findings demonstrate that this trend has not yet been observed in the South African context.

## Conclusion

This study highlights a gap in the current HCV diagnostic algorithm in South Africa. Even with reflex testing having been implemented in South Africa, there are still gaps in patients’ linkage after a positive HCV antibody result. Therefore, if follow-up systems are not strengthened, the benefits of reflex testing are not fully exploited. With a seroprevalence of 4.04% among the general population, our findings underscore the need to frequently update seroprevalence data to inform elimination strategies. Our findings also suggest an ongoing transmission in the general South African population. The lack of follow-up HCV RNA testing highlights a setback in implementing recommended diagnostic algorithms and patients’ linkage to care.

### Limitations of the study

It is important to observe that data from NHLS CDW was used, which reflects testing practices within the South African public health sector only. Clinicians requesting HCV testing in the private sector may have a different approach because of better resources, and therefore, including private sector data may reflect different testing outcomes. Our data could not be stratified by high-risk groups; therefore, we were unable to specifically analyse outcomes for individuals considered to be high-risk groups. Moreover, the seroprevalence findings from routine laboratory data from one province may not be representative of other South African provinces.
